# Convolutional Neural Networks for Image-Based Corn Kernel Detection and Counting

**DOI:** 10.3390/s20092721

**Published:** 2020-05-10

**Authors:** Saeed Khaki, Hieu Pham, Ye Han, Andy Kuhl, Wade Kent, Lizhi Wang

**Affiliations:** 1 Industrial and Manufacturing Systems Engineering, Iowa State University, Ames, IA 50011-3611, USA; lzwang@iastate.edu; 2 Syngenta, Slater, IA 50244, USA; hieu.pham@syngenta.com (H.P.); ye.han@syngenta.com (Y.H.); andy.kuhl@syngenta.com (A.K.); wade.kent@syngenta.com (W.K.)

**Keywords:** corn kernel counting, object detection, convolutional neural networks, digital agriculture

## Abstract

Precise in-season corn grain yield estimates enable farmers to make real-time accurate harvest and grain marketing decisions minimizing possible losses of profitability. A well developed corn ear can have up to 800 kernels, but manually counting the kernels on an ear of corn is labor-intensive, time consuming and prone to human error. From an algorithmic perspective, the detection of the kernels from a single corn ear image is challenging due to the large number of kernels at different angles and very small distance among the kernels. In this paper, we propose a kernel detection and counting method based on a sliding window approach. The proposed method detects and counts all corn kernels in a single corn ear image taken in uncontrolled lighting conditions. The sliding window approach uses a convolutional neural network (CNN) for kernel detection. Then, a non-maximum suppression (NMS) is applied to remove overlapping detections. Finally, windows that are classified as kernel are passed to another CNN regression model for finding the (x,y) coordinates of the center of kernel image patches. Our experiments indicate that the proposed method can successfully detect the corn kernels with a low detection error and is also able to detect kernels on a batch of corn ears positioned at different angles.

## 1. Introduction

Commercial corn (*Zea mays* L.) is processed into numerous food and industrial products and it is widely known as one of the world’s most important grain crops. Based on the United States Department of Agriculture’s yearly results, in 2019, corn added $142.6 billion to the U.S. economy and is estimated to increase to $183.6 billion by 2029 [1]. Corn serves as a source of food for the world and is a key ingredient in both animal feed and the production of bio-fuels [2,3]. In the U.S. approximately 40% of corn is used for ethanol [4] and nearly 49.0% is used to feed animals (pigs, cows, cattle, etc.) [5]. Moreover, the direct use of corn for food worldwide exceeds 150 million tons/year [6]. The importance of corn cannot be understated, and due to the world’s reliance on corn it is imperative that we work to maximize the yield of each ear of corn.

Corn grain yield is driven by optimizing the number of plants per given area and providing sufficient inputs to maximize total kernels per ear within a given environment. Determining corn grain yield is complicated and requires a detailed understanding of corn breeding, crop physiology, soil fertility, and agronomy, but accurate estimates using simple data inputs can provide reliable information to drive certain management decisions. A well developed corn ear can expect to have over 650–800 kernels. However, various environmental stresses can affect corn ear development impacting the total number of kernels per ear. When an ear faces unfavorable environmental conditions, such as drought, heat, moisture, high wind speeds, etc., there is the possibility for the reduction in its yield potential due to the genetic make-up of the corn being vulnerable to theses environmental conditions [7,8]. For instance, drought and heat stress will have a negative correlation with the number of kernels on an ear, due to the fact that some specific types of corn needs more water and cooler climate than others. Moreover, soil fertility limitations and intense pest pressure throughout a growing season can have adverse effects on total kernels developed resulting in lower total grain yield [9,10]. Plant breeders work to maximize the amount of material we gain from corn by breeding existing corn with the most resilient, high-yielding genetics. If total kernels per ear, kernel depth, kernel width and estimated kernel weight can be quickly and accurately measured, additional information could be gathered about the crop and allow farmers to make early accurate management decisions.

### 1.1. Motivation

Precise in-season corn grain yield estimates enable farmers to make real-time accurate harvest and grain marketing decisions minimizing possible losses of profitability [11]. These decision can vary from management practices (applying fungicide, nitrogen, fertilizer, etc.) to determining future holding costs with respect to yield futures from the Chicago Mercantile Exchange [12,13,14]. Due to the manual labor needed to count the number of kernels on an ear of corn, high-throughput phenotyping is not possible due to the necessary manual labor and the possibility of human error. With modern technology, executing yield estimates in real-time digital applications can be done efficiently and consistently, compared to past methods, while providing the ability to make historical comparisons following harvest [15]. Agronomically, accurate in-season yield estimates deliver the unique potential for agronomists and farmers to diagnose potential issues that have or may impact corn grain yield, and equip them with the informed knowledge to make real-time decision with respect to their harvest. Recently, image-processing, machine learning, and deep learning have shown great potential in progressing the digital capabilities needed for the future of agriculture. These techniques have shown to be reliable for high-throughput phenotyping and enable farmers to make real-time decision, something that was previously not possible.

Due to the need to count corn kernels on numerous ears and because of the manual limitation of this task, this work proposes a new deep learning approach to estimating the number of kernels on an ear of corn that can be used for real-time decision making. This methodology takes an image of a single or multiple ears of corn and outputs the estimated number of kernels in the entire image with no assumptions on either the background environment nor the lighting conditions of the image.

### 1.2. Literature Review and Related Works

Succinctly, machine learning is a method of data analysis to automatically identify patterns within data which can be tabular, images, text, etc. The process of machine learning requires building a model on an initial dataset, called the training dataset, and then using an independent dataset, called the test set, to validate the performance of the model on data which was not used for training. This procedure allows for a true representation of the accuracy of the trained machine learning model. There exists a large literature on various machine learning models in a variety of domains [16,17,18,19]. However, we will not provide a review here as ultimately we want to focus our attention on a special case of machine learning often referred to as deep learning.

Deep learning models are representation learning methods with multiple levels of representations. Each level of representations has nonlinear modules to transform the representation at the current level (starting with the raw input) to a slightly more abstract level [20]. Deep neural networks also belong to a class of universal approximators [21], which means regardless of what function we want to learn, they can be used to approximately represent such a function [22]. Deep learning models automatically perform feature extraction on input data without the need of using any handcrafted input of features.

As one of the fundamental components of computer vision, object detection provides information about the concepts and locations of objects contained in each image [23]. As such, the goal of object detection is to localize objects in a given image and determine which category each object belongs to. Traditional object detection methods first extract feature descriptors such as HOG [24] and SIFT [25]. They then train a classifier such as a support vector machine (SVM) [26] and AdaBoost [27] based on extracted feature descriptors to distinguish a target object from all the other categories. More recently, deep learning based object detection methods have been proposed. These methods such as single shot detection (SSD) [28], you only look once (YOLO) [29], and fast R-CNN [30] automatically extract necessary feature descriptors which significantly improves their accuracies compared to traditional object detection methods. However, these methods are very data hungry and computationally expensive to train.

In terms of applying machine learning, image processing, and deep learning for object detection in agriculture, there has been no shortage of use-cases. Traditional image processing based approaches often referred to as image segmentation (filtering, watershedding, thresholding, etc.) have been applied to mangoes, apples, tomatoes, and grapes for detecting and counting within images [31,32,33,34,35,36]. Although, successful, these approaches typically require large amounts of high-resolution images with minimal noise, cannot handle large variation in crop sizes, and can only identify a single crop per image.

Using a machine learning approach, Ok et al. [37] demonstrated that the random forest (RF) algorithm [38] and maximum likelihood classification [39] were indeed suitable at successfully classifying wheat, rice, corn, sugar beet, tomatoes, and peppers within fields using satellite imagery. Additionally, Zawbaa et al. [40] designed an experiment to automatically classify images of apples, strawberries, and oranges using RF and *k*-nearest neighbors model [41]. Their study further demonstrates the success that machine learning capabilities have in agriculture. Moreover, Guo et al. [42] applied a quadratic-SVM [26] to accurately detect and count sorghum heads from unmanned aerial vehicle (drone) images. Although these example show the power that modern machine learning has in object detection, specifically in agriculture, they are not without fault. Namely, tradition machine learning approaches cannot generalize well to objects with varying image resolutions, different image scaling (distance from camera to object) and different object orientations (object angles).

Due to the power of deep learning being able to recognize multiple objects within images and the lack of requirements towards object orientations, there has been a large amount of recent literature in deep learning in agriculture. In 2019, Ghosal et al., applied their method based on a RetinaNet to detect and count sorghum heads from drone images [43]. This deep learning approach significantly outperformed prior sorghum detection and counting work by Guo et al. [42]. Various other deep learning models have also been proposed in disease detection, quality assessment and detection and counting of various crops [44,45,46,47]. DeepCrop is an image repository consisting of 31,147 images with over 49,000 annotations from 31 different crop classes [48]. This dataset has been instrumental in the advancement of object detection in agriculture where often times gathering annotated data is a challenge [49,50]. With the advent of transfer learning, models can be pre-trained on such datasets and have their information transferred to detect similar objects without the need for long training times [51]. Due to the large literature combining deep learning and agriculture, we cannot do justice in providing a comprehensive review. Instead, we point the reader towards a survey paper which gives a thorough overview of image-based plant phenotyping using deep learning [52].

We have provided an overview of image processing, machine learning, and deep learning in various agricultural tasks, as such now we turn our attention to the focus of this paper, namely, work that has been completed in counting corn kernels. In 2014, Zhao et al. [53] applied traditional image processing based approaches to count kernels, but was still limited to the previously mention limitations of requiring high resolution images, low signal to noise ratio, and only being able to count from a single ear per image. Grift et al. [54] also invoked an image processing based approach but limits ear images to be taken within a soft box fitted with controlled and uniform lighting conditions. Moreover, the images in their study contained 360 degree photos, that is, they designed a special lighting box so that lighting conditions were controlled and to take complete photos of the ear. Ni et al., in 2018 [55] and Li et al., in 2019 [56] both utilized deep learning to count corn kernels, however, their algorithms were designed to count kernels already removed from the cob. Although both were able to accurately count kernels, their problem is easier than directly counting kernels while on the ear, due to the distinct spacing between kernels in their images. Additionally, this process does not allow for real-time in-field decision making due to having to shell the kernels off the ear before proceeding with the counting. Although, each of these previous methods have “moved the needle” in regards to kernel counting there is not a concise method which address all of theses limitations.

Due to the difficult nature of this problem and the demand for in-field corn kernel count estimates, we propose a deep learning approach to detect and count corn kernels where kernels are still intact on an ear simply using a 180 degree image. This approach will be robust enough to handle any set of ears regardless of the orientation of the ears and the light conditions present.

## 2. Methodology

**Figure 1:**
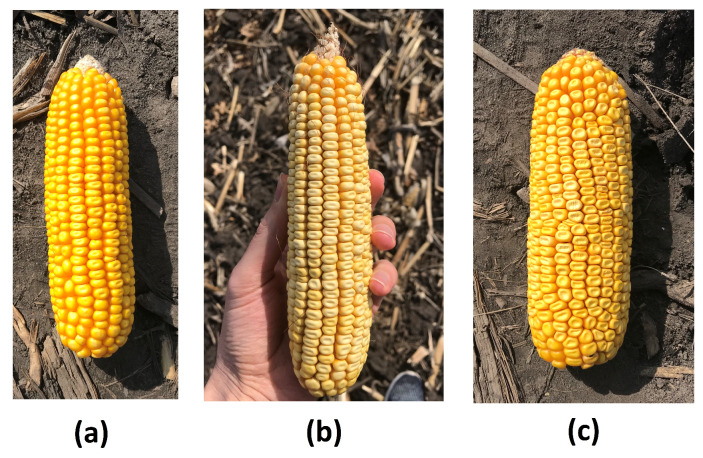
Three genetically different corn ears. Images (**a**–**c**) have different backgrounds. We included different types of backgrounds such as soil, grass, and hands in the training data to make the proposed method robust against the image background.

The goal of this study is to localize and count corn kernels in a corn ear image taken in uncontrolled lighting conditions. To solve this problem, we first detect all kernels in a corn ear image and then estimate the total number of kernels by counting the number of detected kernels. As a result, the underlying research problem is a single class object detection problem. As shown in Figure 1, the number of objects (kernels) in a corn ear is extensive (up to 800 kernels) and the objects are in close proximity to one another, making the problem more challenging.

**Figure 2:**
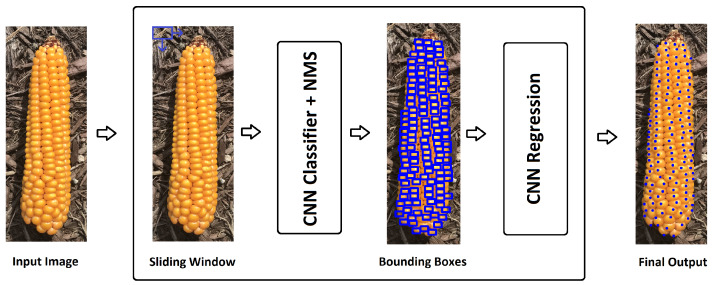
Modeling structure of our proposed corn kernel detection method. A detailed description is given in Section 2.

We use a sliding window approach for kernel detection in this study. At each window position, a convolutional neural network classifier returns a confidence value representing its certainty that the current window contains a kernel or not. After computing all confidence values, a NMS is applied to remove redundant and overlapping detections. Finally, windows that are classified as a kernel are passed to a regression model. The regression model takes in a set of kernel-classified windows which are image patches chosen by the kernel classifier model. Then, each of these selected image patches is fed to the regression model. For example, all kernel-classified windows are shown with blue bounding boxes in Figure 2. The regression model predicts (x,y) coordinates of the center of kernels given image patch of kernels. Figure 2 shows the modeling structure of our proposed corn kernel detection method. Detailed description of the kernel classifier, NMS, and the regression model is provided in the following sections. In this study, we did not use popular object detection methods such as SSD [28], YOLO [29], and fast R-CNN [30] mainly because these methods need considerable amount of annotated images which do not exist publicly for the corn kernel detection. In addition, we could not use transfer learning since corn kernel detection is very different than other object detection tasks such as leaf or human detections.

### 2.1. Corn Kernel Classifier

In this paper, we apply a sliding window approach for kernel detection problem which requires a supervised learning model to classify the current window as either kernel or non-kernel. We use a CNN to classify image patches as CNNs have been shown to be a very powerful method for the image classification tasks [57,58,59,60,61]. The CNN model takes in image patches with size of 32×32 pixels. The CNN architecture for kernel classification is defined in Table 1. All layers are followed by a batch normalization [62] and ReLU nonlinearity except the final fully connected layer which has a sigmoid activation function to produce a confidence value representing the CNN’s certainty that an input image patch contains a kernel or not. Down sampling is performed with average pooling layers. We do not use dropout [63], following the practice in [62].

### 2.2. Non-Maximum Suppression

The kernel classifier outputs a set of candidate proposal bounding boxes for detected kernels. However, these proposal bounding boxes highly overlap and need to be pruned. As such, the non-max suppression algorithm [64], which is a key post-processing step in object detection, is used to remove redundant and overlapping bounding boxes. Let P, S, λ, and D denote the set of initial proposal bounding boxes, set of corresponding confidence scores, overlapping threshold, and set of final proposal bounding boxes, respectively. The non-max suppression algorithm includes the following steps:

1. Select the highest confidence score bounding box from P and add it to D which is initially empty.
2. Remove the selected bounding box from P.
3. Compute the intersection over union (IOU) [65] of the selected proposal box with other proposal boxes in P.
4. Remove all proposal boxes in P which have IOU greater than λ.
5. Repeat the above process until the P is empty.

### 2.3. Regression Model

As shown in Figure 1, the kernels are very close to each other on corn ears. As such, if we visualized all detected kernels with bounding boxes in a corn ear image, it would be almost impossible to see the corn ear, especially on the left and right sides of the ear due to having many close bounding boxes. Furthermore, some kernels have different shapes and angles which might not fit perfectly in a rectangle bounding boxes. As such, we use a convolutional neural network as a regression model which takes in an image of kernel with size of 32×32 pixels and predicts (x,y) coordinates of the center of the kernel. The primary reason for not simply using the center of the windows being classified as kernel as the center of detected kernels is that the center of the kernels are not always in the center of the windows, especially for the kernels on the sides of the corn ear. The CNN architecture for finding the (x,y) coordinates of the center of kernel image is defined in Table 2. All layers are followed by ReLU nonlinearity except the final fully connected layer which has no nonlinearity. Down sampling is performed with max pooling layers. We did not use dropout for this model as it did not improve overall performance. The regression model is applied only on the final windows being classified as a kernel after the NMS. As such, the proposed regression model does not add a lot of computational cost to the kernel detection approach considering the number of final windows being classified as kernel is small.

## 3. Experiments and Results

This section presents the dataset used for our experiments, the training hyperparameters, and the final results. We consider standard evaluation measures such as false positive (FP), false negative (FN), accuracy, and f-score. All our experiments were conducted in Python using the TensorFlow [66] library on a NVIDIA Tesla V100 GPU.

### 3.1. Dataset

**Figure 3:**
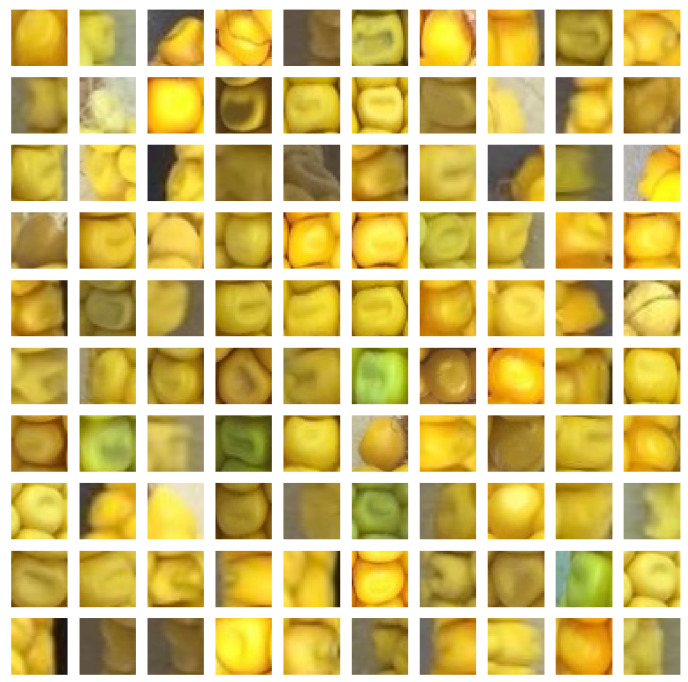
A random subset of kernel images.

**Figure 4:**
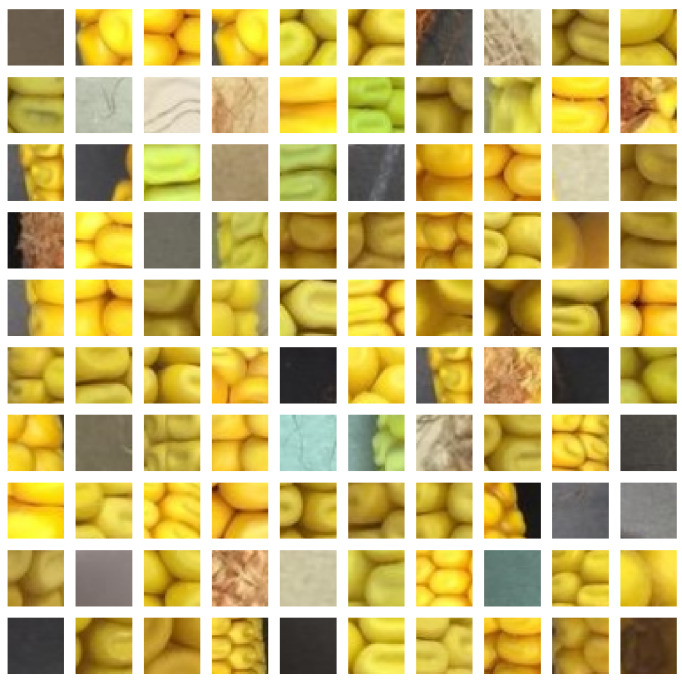
A random subset of non-kernel images.

**Figure 5:**
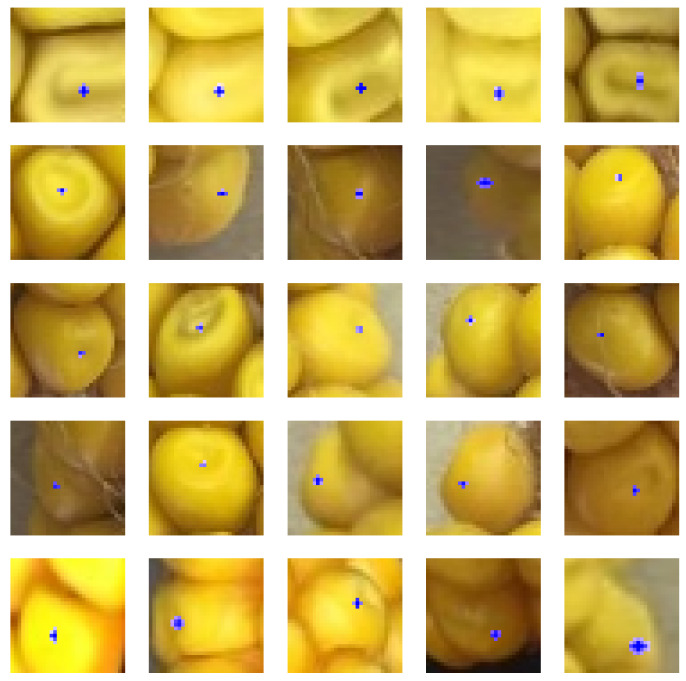
A random subset of annotated kernel images. The blue dot indicates the center of the kernel.

The proposed sliding window approach requires a trained kernel classifier before it can be applied. Therefore, positive samples of kernels and negative samples of non-kernel are necessary. The authors manually cut and labeled kernel and non-kernel images from 43 different corn ear images to generate the training dataset. Each kernel sample is cut out and scaled to 32×32 pixels. Negative samples are generated in the same way using random crops at different positions. The positive samples only include image of one kernel. If the image patch contains two or more kernels, it is considered a negative sample. The training dataset consists of 6978 kernel and 9413 non-kernel samples. Figure 3 and Figure 4 show a subset of kernel and non-kernel images, respectively. For the regression model, we only used the kernel image part of the dataset. We manually labeled the kernel images by finding the (x,y) coordinates of their centers using Labelme [67] software. Figure 5 depicts a subset of annotated kernel images.

### 3.2. Corn Kernel Classifier Training

**Figure 6:**
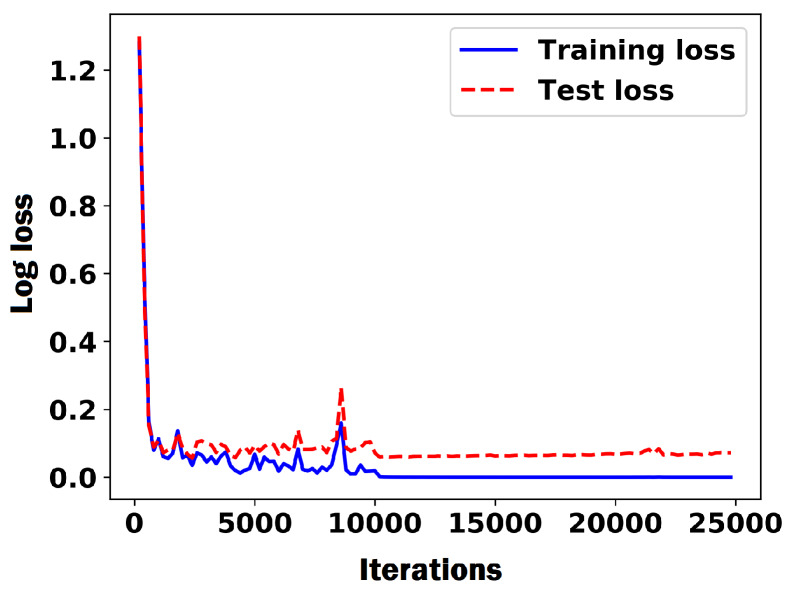
Plot of the log loss of the CNN classifier during training process.

We trained the CNN as described in Section 2.1 for kernel classification using the following training hyperparameters. The weights were initialized with the Xavier initialization [68]. A stochastic gradient descent (SGD) was used with a mini-batch size of 128. The learning rate started from 0.03% and was reduced to 0.01% when error plateaued. The model was trained for 25,000 iterations. Adam optimizer [69] was used to minimize the log loss. For our data, we randomly took 20% of the data as the test data (3278 images) and used the rest as the training data. We augmented around 70% the training data with flip and color augmentations. After augmentation, we had total of 22,292 training images. Figure 6 shows the plot of training and test losses for the CNN. To better evaluate the CNN classifier, a comparison of the CNN classifier with the HOG+SVM model was performed [24]. This model uses the Histogram of Oriented Gradient (HOG) to extract edge features to describe the object’s shape and then trains a support vector machine (SVM) classifier based on the extracted features. The best results achieved for the HOG+SVM were with the parameters 4×4 pixels per cell, 2 cells per block, and 9 histogram bins. Table 3 compares the performances of the CNN and HOG+SVM classifiers on the training and test datasets. We used the CNN model as our final kernel classifier because it resulted in a more reliable kernel detection and counting. Moreover, the CNN model can successfully generalize the prediction to different backgrounds.

Table 3 indicates that the CNN model outperforms the HOG+SVM model with respect to all evaluation measures. One of the reasons for the higher accuracy of the CNN classifier compared to the HOG+SVM is that the CNN automatically extracts necessary features from the data. However, the HOG+SVM model is faster to train and test from computational perspective.

### 3.3. Regression Model Training

**Figure 7:**
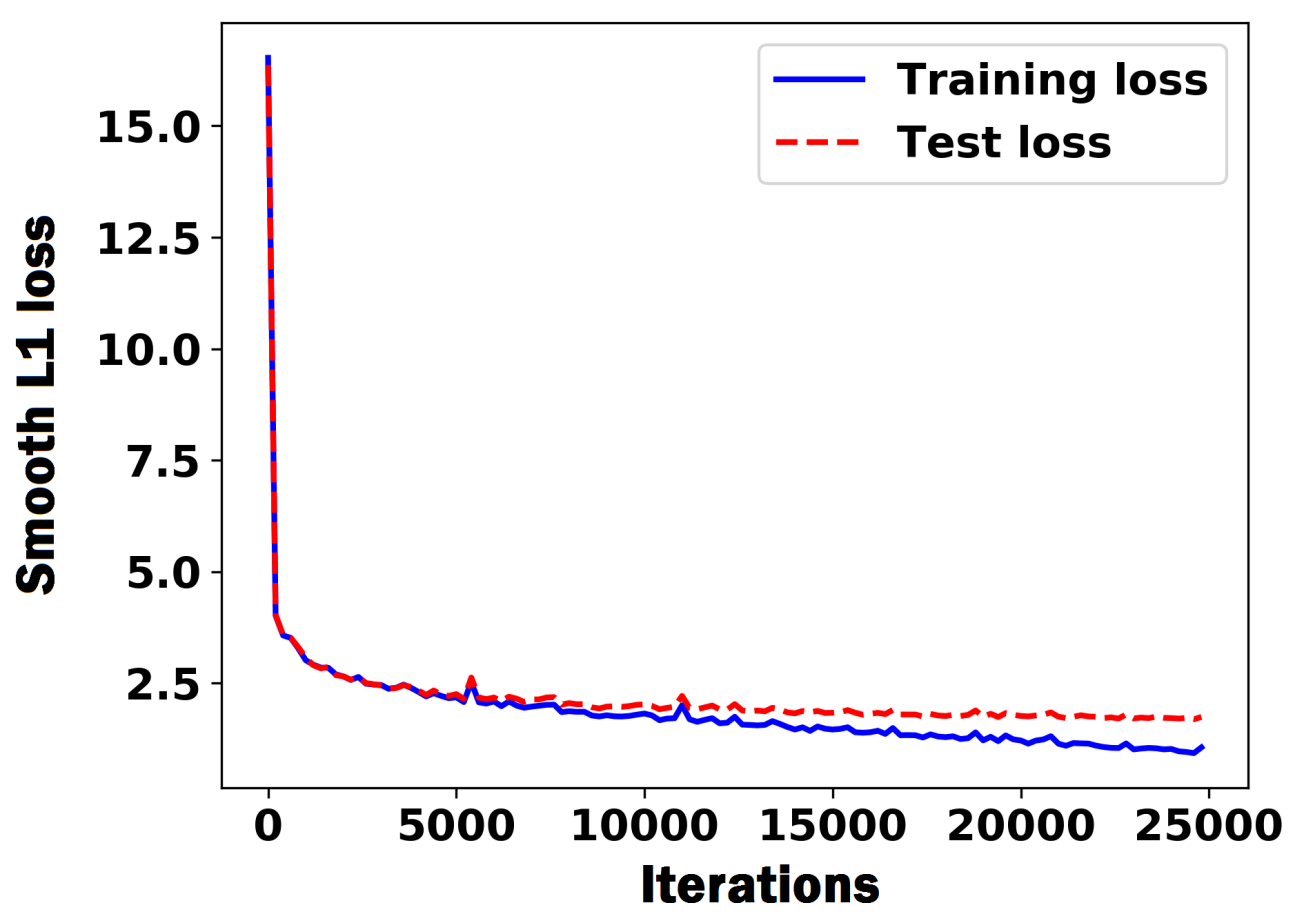
Plot of the smooth $L_{1}$ loss of the CNN regression model during training process. The unit of the loss is pixel.

The CNN model was trained as described in Section 2.3 for finding the (x,y) coordinates of the center of a kernel image using the following training hyperparameters. The weights were initialized with the Xavier initialization. A stochastic gradient descent (SGD) was utilized with a mini-batch size of 45. The model was trained for 25,000 iterations with the learning rate of 0.03%. Adam optimizer was used to minimize the smooth $L_{1}$ loss as in [30], which is less sensitive to the outliers compared to the $L_{2}$ loss. We randomly took 20% of the data as the test data (1,396 images) and used the rest as the training data (5582 images). Figure 7 shows the plot of the training and test losses for the CNN regression model.

### 3.4. Final Results

Having trained our kernel detection model, we can now apply the sliding window approach with the trained CNN classifier on several test images containing full ears. After applying the NMS, the windows that were classified as kernel were passed to the regression model for finding their corresponding centers. We used window size of 32×22 for the sliding window approach. To fully evaluate the proposed approach, we tested the approach on the multiple corn ears with different angles, backgrounds and lighting conditions. Farmers and agronomists assume that corn ears are symmetric [70]. As such, they count the number of kernels on the one side and then double it to approximately find the total number of corn kernels on a corn ear. We used a similar approach except that we multiplied the number of detected kernels on the one side by 2.5 because around 2 columns of kernels on the very left and right sides of the ear are not captured in the image and consequently not counted. The inference time for a corn ear is 5.79 s.

**Figure 8:**
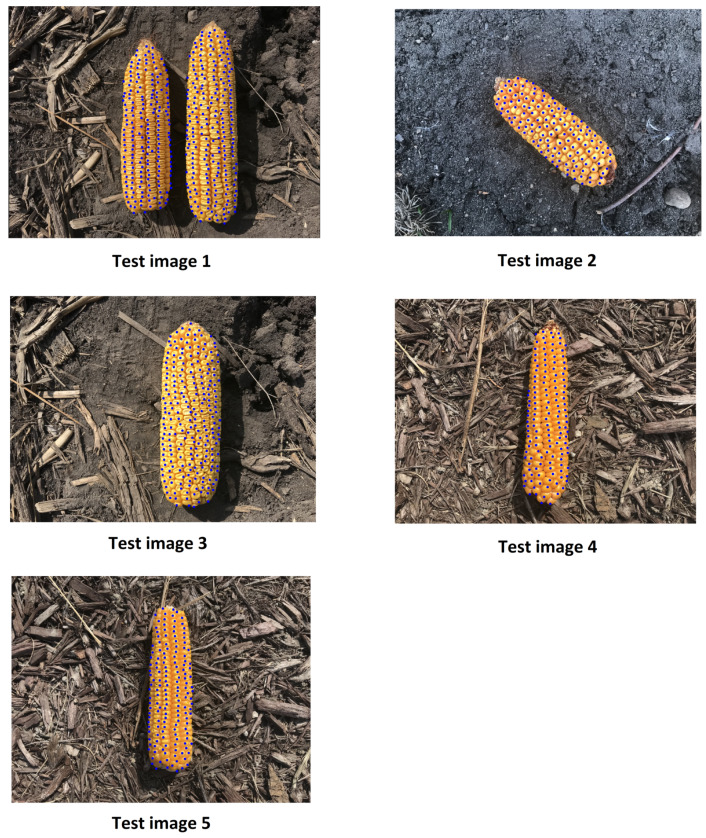
The results of the proposed approach on 5 different test images.

Figure 8 shows the results of the proposed approach on 5 different test images. As shown in Figure 8, the proposed approach successfully found the most of kernels in the test image 1. Test image 2 in Figure 8 shows the results of the proposed approach on the image of an angled corn ear, which was obtained by turning the ear around 45 degrees. Test image 2 is considered a difficult test image because we did not include any angled kernel image in the training dataset. But, the results indicate that the approach can generalize the detection to the images of angled corn ears. We also applied the approach on another difficult test image of a corn ear whose kernels are slightly angled, and as shown in test image 3 in Figure 8, the proposed approach is still able to detect most of the kernels. Test images 4 and 5 in Figure 8 also show the performance of the proposed method on two other test corn ears. Table 4 shows the predicted and the ground truth numbers of the kernels on test images shown in Figure 8. Our proposed approach has the following advantages for kernel counting: (1) our proposed approach can be used on a batch of corn ears, and (2) our proposed approach can be used on a slightly angled corn ear.

To completely evaluate our proposed approach, we manually counted the entire number of kernels on 20 genetically different corn ears and used the proposed method to estimate the number of kernels on these corn ears. We also implemented the method proposed by Chuan et al. [71] called Deep Crowd which was originally developed for people counting in extremely dense crowds using convolutional neural networks. Deep Crowd is one of the state-of-the-art methods proposed for people counting in dense crowds in the literature. The people counting in extremely dense crowds problem is similar to the corn kernel counting problem for two main reasons: (1) they both want to count a large number of objects, and (2) objects are very close to each other. We used the following hyperparameters for training the Deep Crowd method. We used the exact same network architecture as in [71]. We used 43 corn ear images with size of 768×1024 pixels as training data. We randomly cropped 120 patches with 227×227 pixels from each ear image which resulted in the 5160 patches for training the CNN. We also augmented the training data using color and flip augmentations. The CNN was trained using SGD with learning rate of 0.03%.

**Figure 9:**
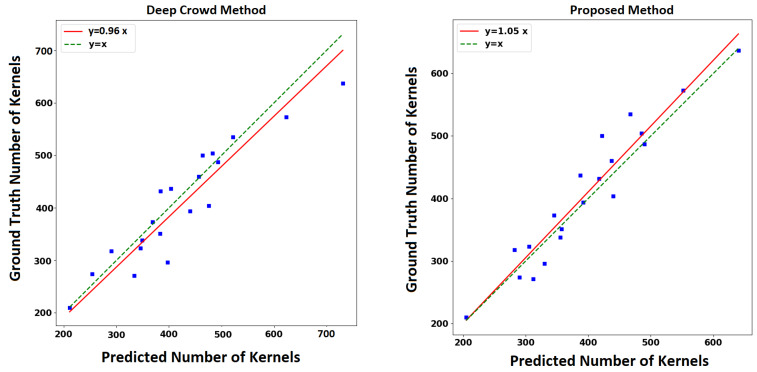
The left and right plots show the predicted number of kernels versus ground truth number of kernels for the Deep Crowd method and proposed method, respectively.

Table 5 compares the performances of the competing methods with respect to the root-mean-squared error (RMSE), mean absolute error (MAE), and correlation coefficient. Figure 9 shows the plot of the estimated number of kernels versus the ground truth number of kernels. The proposed method outperforms the Deep Crowd method with respect to all performance measures. Compared to the Deep Crowd method which only performs counting without localization, the proposed method performs both localization and counting. However, the Deep Crowd method has a smaller inference time compared to our proposed method.

## 4. Discussion

In this paper, we propose a kernel detection and counting method based on the sliding window approach. The proposed method detects and counts kernels on single or multiple corn ears from an image. Compared to the previous studies, the main novelties of our proposed method are summarized as follows: (1) the proposed method detects and counts corn kernel without having to remove the kernels from the corn cob, (2) the proposed method can be used in uncontrolled lighting conditions, (3) the proposed deep learning based method can be utilized without requiring huge amount of annotated images, (4) the proposed method outputs a set of (x,y) coordinates of the center of kernels instead of bounding boxes, which helps better visualize the detected kernels, and (5) our proposed method is also able to detect kernels on a batch of corn ears at different angles.

The sliding window approach uses a CNN classifier for kernel detection. We compared the performance of the CNN classifier with HOG+SVM method. The CNN classifier model performed better than the HOG+SVM method with respect to all evaluation measures because the CNN automatically extracts necessary features from the data which results in a higher prediction accuracy. As such, we selected the CNN model as our final kernel classifier because it resulted in a more reliable kernel detection and counting. In addition, the CNN model can successfully generalize the prediction to different backgrounds.

Moreover, we applied a non-maximum suppression to remove overlapping detections, and finally, windows that are classified as kernel are passed to a regression model for finding the (x,y) coordinates of the center of kernel image patches. We used $L_{1}$ smooth loss for the CNN regression model since we found it to be more robust against the outliers and noises in the data. Due to the effectiveness of the CNN classifier, this approach does not make any assumptions on the lighting conditions, the background quality or the number of ears, or the orientation of the ear like previous approach do. Removing these limitations allows farmers and agronomists to use this in-field to estimate the number of kernels on an ear of corn, given them additional decision making power when it comes to their crop. To evaluate our proposed method, we manually counted the entire number of kernels on 20 genetically different corn ears and used the proposed method and another method called Deep Crowd [71] to estimate the number of kernels on these corn ears. Our proposed method outperformed the Deep Crowd method with respect to all consindered performance measures. The proposed method achieved a RMSE of 8.16% of the average number of kernels for the kernel counting task. We also visualized the detection performance of the proposed method on 5 different test images. As shown in Figure 8, the proposed approach successfully found the most of kernels in the test images. The results suggested that the proposed method can generalize the detection to the images of angled corn ears.

We did not use popular object detection methods such as SSD [28], YOLO [29], and fast R-CNN [30] mainly because these methods need considerable amount of annotated images which do not exist publicly for the corn kernel detection. In addition, we could not use transfer learning since corn kernel detection is very different than other object detection tasks such as car and human detections and features learned from pre-trained models cannot be easily transferred to our kernel detection task. In addition, we included different types of backgrounds such as soil, grass, and hands in the training data to make our proposed method more robust against the image background. This approach could be extended to address several future research directions. For example, similar approach could be used for disease detection and quality assessment of corn.

## Figures and Tables

**Table 1:** The CNN architecture for kernel classification. The Conv, FC, and Avg pool stand for convolutional layer, fully connected layer, and average pooling layer, respectively.

Type/Stride	Filter Size	Number of Filters	Output Size
Conv/s1	3×3	32	30×30×32
Conv/s1	3×3	32	28×28×32
Avg pool/s2	2×2	-	14×14×32
Conv/s1	3×3	64	12×12×64
Conv/s1	3×3	64	10×10×64
Conv/s1	3×3	64	8×8×64
Avg pool/s1	7×7	-	2×2×64
FC-256			
FC-128			
Sigmoid			

**Table 2:** The CNN architecture for finding the (x,y) coordinates of the center of a kernel image. The Conv and FC stand for convolutional layer and fully connected layer, respectively.

Type/Stride	Filter Size	Number of Filters	Output Size
Conv/s1	3×3	32	30×30×32
Conv/s1	3×3	32	28×28×32
Max pool/s2	2×2	-	14×14×32
Conv/s1	3×3	64	12×12×64
Conv/s1	3×3	64	10×10×64
Conv/s1	3×3	64	8×8×64
Max pool/s2	2×2	-	4×4×64
FC-100			
FC-50			
FC-10			
FC-2			

**Table 3:** Performance comparison of the CNN and HOG+SVM classifiers on the training and test datasets.

Classifier		Evaluation Measures			
		FP	FN	Accuracy	F-Score
Training	HOG+SVM	596	595	0.947	0.937
	CNN	0	0	1.0	1.0
Test	HOG+SVM	135	135	0.918	0.906
	CNN	19	22	0.987	0.985

**Table 4:** The predicted and the ground truth numbers of the kernels on test images shown in Figure 8.

Test Image	Predicted Number of Kernels	Actual Numbers of Kernels
1	1012	1046
2	312	323
3	550	585
4	342	296
5	390	394

**Table 5:** The performances of the competing methods on the kernel counting task of 20 different corn ears.

Method	RMSE	MAE	Correlation Coefficient
Proposed	33.11	25.95	95.86
Deep Crowd [71]	45.29	35.25	93.12
